# Notice of Retraction: Interventions That Affect Gastrointestinal Motility in Hospitalized Adult Patients: A Systematic Review and Meta-Analysis of Double-Blind Placebo-Controlled Randomized Trials

**DOI:** 10.1097/MD.0000000000006276

**Published:** 2017-02-24

The publisher has requested that the article “Interventions That Affect Gastrointestinal Motility in Hospitalized Adult Patients: A Systematic Review and Meta-Analysis of Double-Blind Placebo-Controlled Randomized Trials”,^[1]^ which appeared in Volume 95, Issue 5 of *Medicine*, be retracted. The article states in the Methods section that the analysis was done on adult in-hospital patients. However, the studies analyzed used outpatient cohorts. Although this may not have affected the outcome of the study, the methods were misrepresented.
